# Papillary Renal Cell Carcinoma: Demographics, Survival Analysis, Racial Disparities, and Genomic Landscape

**DOI:** 10.15586/jkcvhl.v10i4.294

**Published:** 2023-12-26

**Authors:** Asad Ullah, Abdul Qahar Khan Yasinzai, Naema Daino, Bisma Tareen, Zulfiqar Haider Jogezai, Haleema Sadia, Nimra Jamil, Girahnaz Baloch, Adil Karim, Kaleemullah Badini, Agha Wali, Abdul Waheed, Marjan Khan, Bina Asif, Kaleemullah Kakar, Saleh Heneidi, Feroze Sidhwa, Nabin R Karki

**Affiliations:** 1 Department of Pathology and Laboratory Medicine, Texas Tech University Health Sciences Center, Lubbock , TX, USA;; 2 Department of Medicine, Bolan Medical College, Quetta, Pakistan;; 3 Medical College of Georgia, Augusta, GA, USA;; 4 Aga University Medical College Pakistan;; 5 Khyber Medical College, Pakistan;; 6 Department of Internal Medicine, Integris Baptist Health, Oklahoma City, OK, USA;; 7 Department of Surgery, San Joaquin General Hospital, French Camp, CA, USA;; 8 Department of Internal Medicine, Marshfield Clinic, Marshfield, WI, USA;; 9 Bannu Medical College, Bannu, Pakistan;; 10 Department of Pathology, Kaiser Permanente South Bay, Los Angeles, CA, USA;; 11 Department of Hematology and Medical Oncology, University of South Alabama, Mobile, AL, USA

**Keywords:** COSMIC, next-generation sequencing, papillary renal cell carcinoma, SEER

## Abstract

Papillary renal cell carcinoma (PRCC) is the second most common histological subtype of renal cell cancer. This research aims to present a large database study highlighting the demographic, clinical, and pathological factors, racial disparities, prognosis, and survival of PRCC. The clinical and demographic data were extracted from the Surveillance, Epidemiology, and End Results (SEER) database, and molecular data was cured from the Catalogue Of Somatic Mutations in Cancer (COSMIC) database. PRCC had a median age of diagnosis at 64 years, with a higher incidence in men (77%), and Whites (68%). 70.3% of cases were Grades I–IV (13, 53, 31, and 3%, respectively). In patients with known data, 85% were localized to the kidney, and 84% of cases were 7 cm in size. No metastasis occurred in 97% of the known data. The most common treatment offered was surgical resection (9%). The 5-year overall survival was 79%, with patients undergoing surgery having a 90.6% 5-year survival. Multivariable analysis revealed age > 60 years, Black race, poor histologic differentiation, distant metastases, and tumor size > 10 cm as independent risk factors for mortality. The most common mutations identified from the COSMIC database were MET, KMT2D, KMT2C, ARID1A, and SPEN. PRCC affects male individuals in the sixth decade of life. Increased age, Black race, distant metastases, and tumors > 10 cm are associated with a worse prognosis. Surgical resection offers a favorable survival outcome. Next-generation sequencing (NGS) could identify potentially targetable alterations and future personalized therapeutic approaches.

## Introduction

Renal cell carcinomas (RCC) are subdivided into clear cell RCC and non-clear cell RCC. Non-clear cell RCC comprises papillary RCC (PRCC), chromophobe RCC, oncocytic RCC, and collecting duct carcinoma (1). Histologically, PRCC is differentiated from clear cell RCC by the existence of basophils or eosinophils in the papillary or tubular structures of the kidney (2). Papillary RCC is the second most common RCC, accounting for 10–15% of all RCC cases. The peak incidence is in the sixth to eighth decades of life, with males being approximately twice as likely as females to develop PRCC.

Asian American and Pacific Islander patients have the lowest incidence of renal cancer in the United States when compared to patients of other ethnicities. African Americans and White American patients had a similar 5-year survival rate (approximately 75%). Risk factors included patients with end-stage renal disease on hemodialysis, hypertension, obesity, smoking, male sex, and family history (2–4). Traditionally, PRCC has been classified as Types 1 and 2. Type 1 PRCC is associated with chromosomes 7 and 17 gains, while Type 2 PRCC is more heterogenous with gains in chromosomes 12, 16, and 20 (4). The divisions were based on morphology as Type 1 PRCC has slender papillae lined by a single layer of small basophilic cells and Type 2 PRCC has broader papillae, multiple layers of eosinophilic cells, nuclear pseudo stratification, and larger nucleoli (4, 5). In 2022, the World Health Organization (WHO) classification of PRCC adapted to focus on phenotypes and molecular traits as Types 1 and 2 mixtures were common (5). The recent reclassification of PRCC integrating morphology, immunophenotype, and molecular analysis delineates newer subtypes such as biphasic squamous alveolar RCC (bearing MET mutations), biphasic hyalinizing psammomatous RCC (bearing NF2 mutations), papillary renal neoplasm with reverse polarity (bearing chromosomes 7 and 17 gains, GATA3 and L1CAM nuclear positivity, and KRAS mutations), Warthin-like PRCC, and thyroid-like follicular RCC (5–8).

Clinical features of PRCC include weight loss, flank pain, palpable renal mass, hematuria, fatigue, fever, and night sweats although many patients are usually asymptomatic in the early stages (9). While renal masses can be identified on ultrasound, the first-line modalities for diagnostics are abdominal CT or MRI (with IV contrast) usually revealing homogeneous solid masses (10–11). At the time of diagnosis, PRCC tumors are more likely to be localized. If metastases occur, it favors the lung, bone, liver, and brain, and have a high preference for lymph node involvement. Localized PRCC confined to a kidney is managed with partial or radical nephrectomy, ablation, or active surveillance. There is a 5-year recurrence-free survival (RFS) of 95.8% and a 10-year RFS of 73% with patients undergoing partial nephrectomy (2). Treatment for advanced PRCC involves checkpoint inhibitor immunotherapy, vascular endothelial growth factor receptor (VEGFR) inhibitors, and/or molecular targeted therapies. Management of PRCC in patients with advanced, nonresectable, or metastatic disease is through palliative care (2–3).

This study aims to provide one of the largest and most up-to-date database studies aimed at investigating the demographic, clinical, and pathological factors affecting the prognosis and survival of patients with PRCC.

## Material and Methods

The Surveillance, Epidemiology and End Results (SEER) database initiated by the National Cancer Institute in 1973 covers approximately 28% of the US population. The SEER*Stat software (Version 8.4.0) was used to collect data from 2000 to 2018 using the International Classification of Diseases version 3 (ICD-O-3) and anatomical (C64.9 and C65.9) and histological codes (8260/3). The data was extracted from 18 registries of the SEER database by using SEER software 8.4.0 (https://seer.cancer.gov/seerstat/. Accessed on: 20 January 2023). We also evaluated the most common genetic mutations in the Catalogue of Somatic Mutations in Cancer (COSMIC) (https://cancer.sanger.ac.uk/cosmic. Accessed on: 25 January 2023). Of note, the COSMIC database is independent of the SEER database, and this information is not available on the SEER database.

The data was exported to Statistical Analysis System (SAS) version 9.4 for demographics, univariable tests of associations, proportional hazard model, and non-parametric survival analysis. For proportional hazard cox regression, the univariate test was run between age, gender, race, grade, size, and stage to screen variables for the multivariate model with an accepted P-value <0.25. The multivariate model was then run to identify significant variables associated with mortality. Demographic and clinical data included age, race, tumor grade, tumor size, metastasis, surgical treatment, radiotherapy, chemotherapy (includes all systemic treatment), overall survival, and survival with surgery, radiation therapy, and chemotherapy. The cases included those which were microscopically confirmed “positive histology,” and/or positive genetic studies. All those cases were added where the data was collected “type of reporting resource” from hospital inpatient or outpatient or clinic, laboratory only (hospital or private), physician’s office or private medical practitioner (LMD), nursing or convalescent home or hospice. The cases which are excluded from our study were positive laboratory test or marker study, direct visualization without microscopic confirmation, radiography without microscopic confirmation, clinical diagnosis only, and those cases with unknown status. The cases “type of reporting source” autopsy only, death certificate only were also excluded from the analysis. For survival analysis, those cases are included which are microscopically confirmed, malignant behavior, and patients with known age. The cases excluded from the survival analysis software were, all death certificates only and autopsy only, alive with no survival time.

## Results

In total, 23803 cases of PRCC were identified from 2000 to 2018 in the SEER database. A total of 431 samples were analyzed from PRCC for mutations in the COSMIC database.

### Demographic data and tumor characteristics

The median age was found to be 64 years with a standard deviation (SD) of 11.8 years. Most were aged < 70 years, that was around 16,670 (70%) cases. 76.7% of the cases were males. The majority of the cases were Whites (68.5%) by race. The grade of 70.3% of the cases were known, of which 52.7% were of Grade II. 84.6% of the cases were localized (to the kidneys). The tumor size was ≤7 cm in 83.6% of the cases when known (Table 1).

**Table 1:** Demographic profiles and tumor characteristics of papillary renal cell carcinoma.

	Variable (n = 23803)	Frequency (%)
Age (years)	<70	16,670 (70%)
	≥70	7133 (30%)
Gender	Female	5547 (23.3%)
	Male	18,256 (76.7%)
Race	Unknown	151 (0.6%)
	White	16,311 (68.5%)
	Black	6557 (27.5%)
	Asian or Pacific Islander	683 (2.9%)
	American Indian or Alaska Native	101 (0.4%)
Grade	Unknown	7060 (29.7%)
	Known	16743 (70.3%)
	Grade where known (n = 16,743)	
	Well-differentiated; Grade I	2156 (12.9%)
	Moderately differentiated; Grade II	8831 (52.7%)
	Poorly differentiated; Grade III	5193 (31.0%)
	Undifferentiated; anaplastic; Grade IV	563 (3.4%)
Stage	Unknown	2150 (9.0%)
	Known	21,653 (91.0%)
	Stage where known (n = 21,653)	
	Localized	18,326 (84.6%)
	Regional	2279 (10.5%)
	Distant	1048 (4.9%)
Size	Unknown	7949 (33.4%)
	Known	15,854 (66.6%)
	Size where known (n = 15854)	
	(0 cm) No tumor found/Microscopic (<1mm)	17 (0.1%)
	≤7 cm	13,254 (83.6%)
	7.1–10 cm	1417 (8.9%)
	>10 cm	1166 (7.4%)
Laterality	Right – origin of primary	11,830 (49.7%)
	Left – origin of primary	11,855 (49.8%)
	Bilateral – single primary	31 (0.1%)
	Paired site – but no information concerning laterality of primary	70 (0.3%)
	Only one side – side unspecified	17 (0.1%)

### Treatment characteristics and distant metastasis at the time of diagnosis

**Figure 1:**
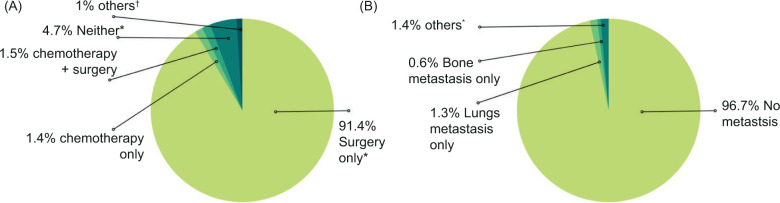
Papillary renal carcinoma, (A) treatment modalities, (B) metastatic pattern at the time of diagnosis.

Of the total cases, the chemotherapy statuses were unknown in 91.4% of the cases, but they underwent surgery without radiation. Only 1.4% of the cases had chemotherapy (any systemic therapy) (Figure 1A).

The site of metastases at presentation was unknown in 37% of the cases and was known in 63.0% of the cases. There were no metastases in majority (96.7%) of the known cases. The most common site of metastasis was the lungs (1.3%) (Figure 1 B).

### Outcomes and survival analysis

**Figure 2:**
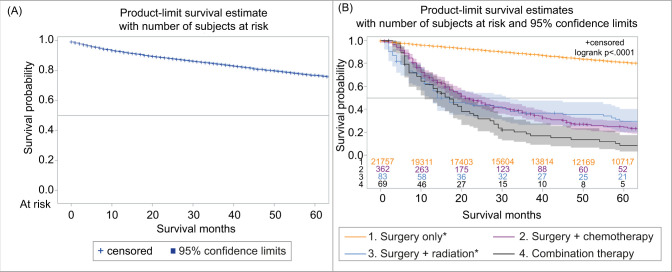
Papillary renal cell carcinoma, (A) overall survival, (B) survival with different treatments.

**Figure 3:**
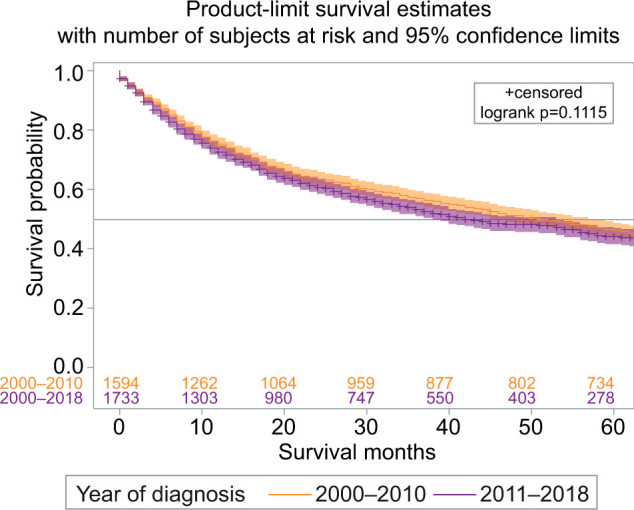
Regional and distant stage papillary renal cell carcinoma survival difference among those diagnosed between 2000 and 2010, and 2011 and 2018.

**Figure 4:**
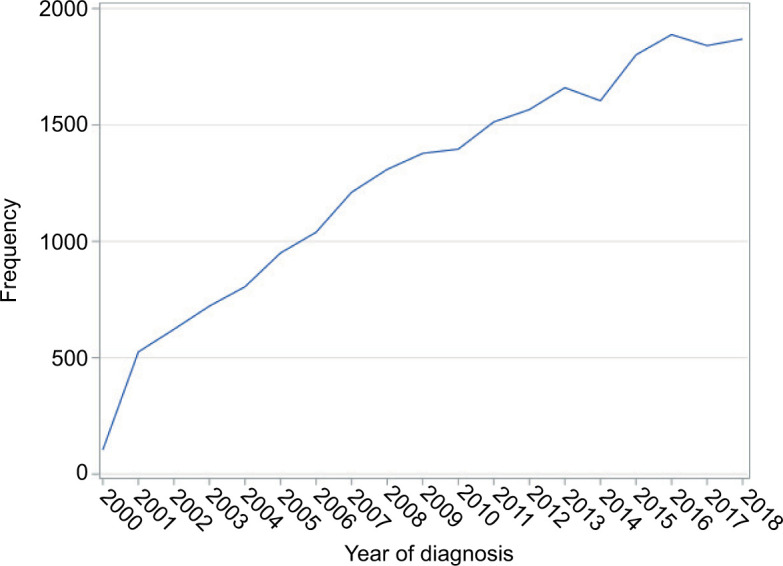
Papillary renal cell carcinoma annual trend from 2000 to 2018.

The overall 5-year survival was observed in 76.6% of cases with a 95% confidence interval (CI, 77.9–79.3), and 5-year cause-specific survival (CSS) was observed in 88.2% (95% CI, 87.7–88.8) (Figure 2A). The 1-year and 5-year survival with chemotherapy (all systemic therapies) were 58.3 (95% CI, 54.1–62.2), and 17.1% (95% CI, 17.7–20.7), respectively. The survival rates at 1 year and 5 years in patients who underwent surgical resection of the tumor were 97.2 (95% CI, 96.9–97.4) and 90.6% (95% CI, 90.1–91.1), resepectively. The survival of patients with surgery and chemotherapy at 1 year was 69.7% (95% CI, 64.5–74.3), and at 5 years was 23.5% (95% CI, 18.5–28.7). The 1-year and 5-year survival with radiation therapy were 57.1 (95% CI, 48.3–65.0) and 20.4% (95% CI, 13.8–28.0), respectively. The survival with combination therapies (surgery and adjuvant chemoradiation) at 1 year was 62.4% (95% CI, 48.0–73.9), and at 5 years was 11.4% (95% CI, 4.3–22.2). The best overall long-term survival was observed with surgery followed by surgery and adjuvant radiation, whereas the combination therapies (surgery and adjuvant chemoradiation) showed no significant improvement in survival (P < 0.0001) (Figure 2B). In regional and distant-stage diseases, there were no significant differences in survival in those that were diagnosed between 2000 and 2010, and 2011 and 2018 (P = 0.1115) (Figure 3). The cases of PRCC is consistently spiking since 2000 (Figure 4). The increase in the number of cases may be related to the improvement of diagnostic modalities over the period of 18 years.

### Survival analysis by race and gender

The survival in males at 1 year was 95.2% with a 95% CI (94.8–95.6), and 5-years survival was 88.0% (95% CI, 87.4–88.6). The survival in females at 1 year was 95.6% (95% CI, 94.9–96.2) and at 5 years was 88.9% (95% CI, 87.7–89.9). There was no survival benefit observed for gender.

**Figure 5:**
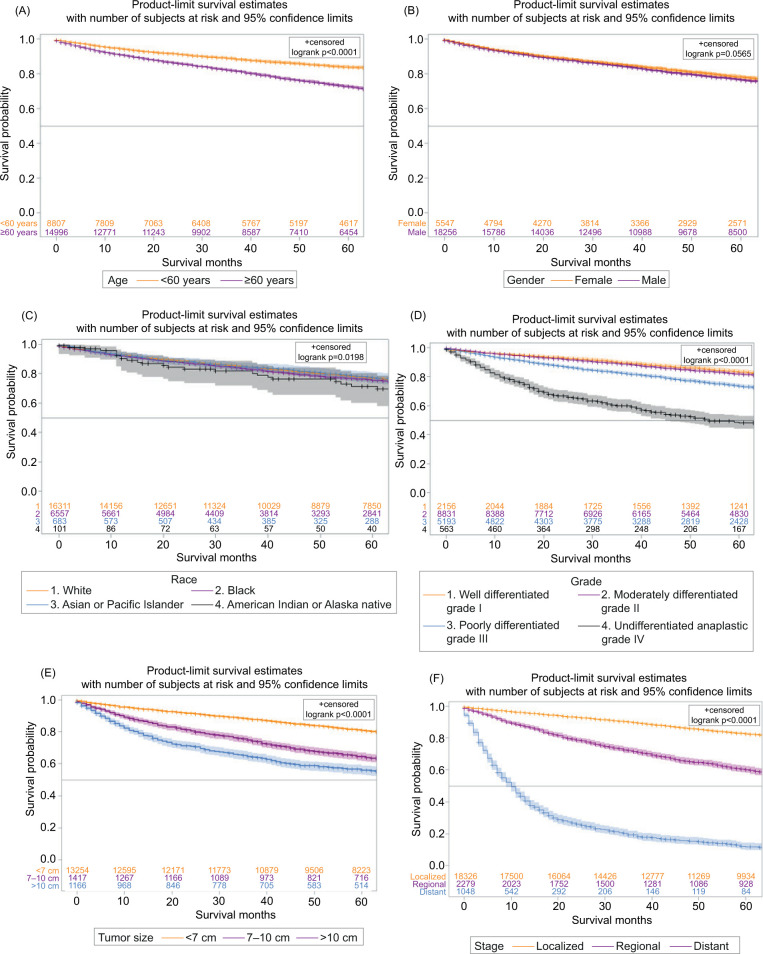
Papillary renal cell carcinoma, (A) overall by Age (for the age cutoff was <60 years and >60 years), (B) survival by gender, (C) survival by race, (D) survival by tumor grade, (E) survival by tumor size, (F) survival with tumor stage.

The 1-year survival for White Americans was 95.3% with a 95% CI (94.9–95.7), and at 5 years the survival was 88% (95% CI, 87.6–88.9). For Blacks, 1-year survival was 95.6% with a 95% CI (95.0–96.2), and 5 years was 88.7% (95% CI, 87.1–89.7). Survival analysis for Asians or Pacific Islanders at 1 year was 94.1% with a 95% CI (91.7–95.9), and at 5 years was 83.1% (95% CI, 79.0–86.5). The survival for American Indians or Alaska natives at 1 year was 94.8% (95% CI, 86.7–98.0) and 5-years survival was 86.7% (95% CI, 75.6–92.9). Age > 60, Grade IV, tumor size > 10, and distant disease were associated with worse clinical outcomes (P < 0.0001) (Figure 5).

### Racial disparities

The median age at diagnosis was 65 years for White and 60 for Black race. The percentage of cases with age > 60 was higher in Whites 11,063 (67.8%) than in Blacks 3374 (51.5%). The percentage of Grade IV cases were slightly higher in Whites (403, 2.5%) than in Blacks (139, 2.1%). The percentage of distant stage was slightly higher in Whites (722, 4.4%) than in Blacks (273, 4.2%). The percentages of both bone and brain metastases were almost similar in both races; 152 (0.9%) and 31 (0.2%) in Whites, and 59 (0.9%) and 13 (0.2%) in Blacks, respectively. Most of the percentages were higher in White race; however, the percentage of tumor size >10 cm was higher in Blacks (380, 8.7%) than Whites (725, 6.7%). The percentage of liver and lungs metastases were slightly higher in Blacks, 47 (0.7%) and 90 (1.4%), than in Whites 78 (0.5%) and 211 (1.3%), respectively. The chi-square test of association found all these correlations significant (P < 0.05) (Table 2).

**Table 2:** Racial disparity table of 23803 patients with papillary renal cell carcinoma.

Variables	Race frequency (% by total individual race)	
	White	Black
Median age at diagnosis	65	60
Age > 60 years	11,063 (67.8%)	3374 (51.5%)
Grade IV	403 (2.5%)	139 (2.1%)
Distant metastases	722 (4.4%)	273 (4.2%)
Tumor size >10 cm	725 (6.7%)	380 (8.7%)
Bone metastases	152 (0.9%)	59 (0.9%)
Brain metastases	31 (0.2%)	13 (0.2%)
Liver metastases	78 (0.5%)	47 (0.7%)
Lung metastases	211 (1.3%)	90 (1.4%)
Mean survival time (months)	67.9	62.1
Dead events	4887 (30.0%)	1918 (29.3%)

### Multivariable analysis

Multivariable analysis through Cox survival regression analysis identified age > 60 years hazard ratio, HR 2.096 (P = 0.001); Black race HR 1.178 (0.001); undifferentiated, anaplastic, Grade IV HR 1.785 (P = 0.001); distant stage HR 9.694 (P = 0.001); and size > 10 cm HR 1.424 (P = 0.001) as factors associated with increased mortality, P < 0.05 (Table 3).

**Table 3:** Multivariate analysis of independent factors influencing mortality papillary renal cell carcinoma.

Variables		Multivariate analysis; Hazard ratio (P)
Age	>60	2.096 (0.001)
Race	Black	1.178 (0.001)
Grade	Undifferentiated; anaplastic; Grade IV	1.785 (0.001)
Stage	Distant	9.694 (0.001)
Size	>10 cm	1.424 (0.001)

### Mutational analysis of papillary renal cell carcinoma from the COSMIC database

The data for genetic mutations from Catalogue of Somatic Mutations in Cancer (COSMIC) (https://cancer.sanger.ac.uk/cosmic. Accessed on: 25 January 2023) version GRCh37 COSMIC v97 was extracted, and a total of 6832 cases was evaluated for genetic mutations. Of these mutations, 5116 cases were categorized as carcinoma. In sub-histology selection criteria for papillary RCC, a total of 431 cases were evaluated for genetic mutations. The top 20 genes mutated in papillary RCC were MET (9%), KMT2D (6%), KMT2C (5%), ARID1A (5%), SPEN (5%), FAT11 (5%), KIT (4%), SETD2 (4%), LRP1B (4%), SMARCA4 (4%), NF1 (4%), ARID1B (4%), KDM6A (3%), NF2 (3%), NFE2L2 (3%), BAP1 (3%), ATM (3%), NCOR (3%), ZFHX3 (3%), and VHL (2%).

COSMIC database revealed alterations in MET in 63 of 709 (9%) PRCCs tested for missense mutations. There were 14 copy number gains identified from the 286 (4.8%) tested. MET missense mutations were seen in one out of seven copy number gains.

## Discussion

This study includes one of the largest groups of patients with PRCC. The Median age of diagnosis was 64 11.8 years, and a higher incidence in men (76.7%) was noted. The majority of cases were localized (84.6%) with no metastases in 96.7% of the known cases.

The cases of PRCC is consistently increasing since 2000 to 2018, likely reflecting better diagnostics and improved screening guidelines. Per previous reports, PRCC is prevalent in the sixth to eighth decades of life and has a male sex preference with a male-to-female ratio of 1.5–2:1 (2, 12). Mendhiratta et al. (2) reported that there are roughly three times as many Black patients diagnosed with PRCC with relative to other races. Our analysis showed a frequency of PRCC in 27.5% of Black patients and a frequency of 68.5% in White patients. It is important to note that our study included a larger number of White participants (16,311) compared to Black participants (6557). In patients where tumor metastasis was known, 96.7% reported no metastasis. If metastasis occurred, common locations included the bone, brain, liver, and lungs. These findings are consistent with what has been reported in the literature (2). The overall observed 5-year survival rate was recorded as 78.6%, and previous studies reported a value of 61% (13).

The goals of therapy are dependent on the extent of the disease. Treatment options for localized PRCC include surgical removal (partial or radical nephrectomy), ablation (cryoablation, radiofrequency ablation, or microwave ablation), or active surveillance, similar to the treatment options for localized clear cell RCC (2, 12). Small PRCC (4 cm) confined to the kidney can be managed with nephron-sparing surgery (partial nephrectomy), as it produces satisfactory patient outcomes (12, 14). Radical nephrectomy may be performed depending on the extent of disease, tumor location, presence of metastases, as well as additional patient-specific factors including patient comorbidities and overall health status. Surgical resection is typically the definitive therapy for localized disease without metastases as it offers the greatest chance of cure (12, 14, 15).

Treating advanced PRCC takes into consideration histopathology, molecular variations, and clinical presentations as these characteristics play a role in therapy response. Most of what is known about treating advanced PRCC comes from our understanding of clear cell RCC (16). Systemic therapy is typically administered as a single therapy or in combination with other agents. Available therapies include programmed cell death protein 1 (PD-1) checkpoint inhibitors (nivolumab and pembrolizumab), programmed cell death ligand 1 (PD-L1) checkpoint inhibitors (atezolizumab), anticytotoxic T lymphocyte–associated protein 4 (CTLA-4) antibody (ipilimumab), antiangiogenic therapy also known as vascular endothelial growth factor (VEGF) inhibitors (sunitinib, cabozantinib, axitinib, lenatinib, bevacizumab), mammalian target of rapamycin (mTOR) inhibitors (everolimus and temsirolimus), experimental MET inhibitors (crizotinib, foretinib, and savolitinib), and rarely cytotoxic chemotherapy (12, 17–23).

Although recent WHO reclassifications have a single PRCC category, existing literature classifies PRCC into Types 1 and 2, primarily based on histopathology (12, 16, 24). Type 1 PRCC is characterized by basophilic cells, small nuclei, and a single layer of cuboidal or columnar epithelial cells (2). It is typically confined to the kidney and is associated with a good prognosis. Type 1 PRCC is associated with hereditary renal cancer syndrome (germline MET) and gains in chromosomes 7 and 17 along with gains in chromosomes 2, 3, 12, 16, and 20 that occur less often (12, 18, 20, 25). Therapeutic agents such as crizotinib, savolitinib, and cabozantinib show promising inhibitor properties against MET, which are useful in treating type 1 PRCC (20). Type 2 PRCC is characterized by eosinophils, psuedostratification, and prominent nucleoli with an unfavorable prognosis as it has an aggressive disease course (2, 20). These tumors are less likely to have MET mutations, present at an advanced stage, and run an aggressive course. It may be associated with hereditary leiomyomatosis and renal cell carcinoma (HLRCC) syndrome; germline fumarate hydratase (FH)) (18, 20). A focal loss of 9p21 led to a loss of CDKN2A has also been identified in patients with Type 2 PRCC. Poor overall survival is the consequence of patients with mutations in CDKN2A (26). Erlotinib + bevacizumab is the treatment choice for HLRCC (27, 28).

PRCC also displays mutations in previously studied cancer-related pathways; these include NF2 (Hippo pathway), SMARCB1 and PBRM1 (SWI/SNF complex), and chromatin modifier pathways (SETD2, KDM6A, and BAP1). Genes in these pathways had high mutation rates in Type 1 and Type 2 PRCC in previous reports. Mutations involving SWI/SNF complex accounted for 20% of mutations in Type 1 and 27% of mutations in Type 2. Chromatin modifier pathways showed alterations in 35% of Type 1 and 38% of Type 2 PRCC. The Hippo pathway revealed mutations in 3% of Type 1 and 10% of Type 2 (25). Tumor clusters have also been identified as part of the pathogenesis of PRCC. Cluster C1 is similar to Type 1 PRCC as it exhibits MET mutations and chromosome 7 gains. Cluster C1 is associated with a better prognosis. Clusters C2a and C2b are similar to Type 2 PRCC. Cluster C2c has a CpG island methylator phenotype (CIMP), which was revealed to have the lowest survival probability (25).

Response to the available therapies is limited in both advanced Type 1 and Type 2 PRCC. Although the treatment approaches are extrapolated from that of clear cell RCC, the response and outcomes do not have the same robust level of evidence (16). In all, treatments have yet to be standardized for advanced diseases. Treatment rather focuses on molecular characterization to provide individualized treatment options to patients (12). Although previous studies have suggested that PRCC has a better prognosis than clear cell RCC, recent studies show this may not be the case as the long-term prognoses were comparable (29). In localized PRCC, surgical resection is favorable to patient outcomes with a reduced metastatic ability compared to clear cell RCC. Advanced PRCC with metastases has a poor prognosis as it has a poor response to therapy due to less effective treatments and the development of resistance (30). While earlier studies showed Type 2 as more aggressive and with more unfavorable outcomes than Type 1, more recent studies have shown that the subtype is not an effective predictive measure of patient survival (31, 26).

Activations in MET can occur through an activating MET splice site alteration resulting in the skipping of exon 14 or in amplification or copy number gain (CNG) of the chromosomal region harboring MET, at the 7q31 locus of chromosome 7 (32–34). MET analysis was extensively reviewed, showing 43 cases of MET-altered PRCC with reported CNG, 15 were whole chromosome gain of 7. These were likely to be less responsive to targeted MET inhibition, although rare cases have been reported (35). MET alterations generally impacting the tyrosine kinase domain (amino acid range 1149–1248) have been associated with constitutive activation and are commonly linked to PRCC Type 1 histology, characterized typically as non-aggressive (36). With advancements in clinical trial data concerning immune checkpoints, the standard systemic therapy for PRCC has shifted. Agents targeting the MET proto-oncogene in PRCC have produced promising results; however, these advancements need to be further studied to discern if these targeted therapies have meaningful impact (37). Our study did not find any significant survival difference in those that were diagnosed before 2010 compared to those that were diagnosed after 2010 despite the improvement in therapies, warranting further research and better treatment options for advanced disease.

## Limitations

Using a sizable national database, our study aims to describe the clinical and demographic characteristics of patients with PRCC. Our study has similar limitations that are common to many database studies. First, the SEER database does not provide important information for factors such as comorbidities, and associated renal pathologies that could influence the interpretation of results. Second, the SEER database did not include information on the treatment plan and stage-specific management. Additionally, the SEER database does not contain information on the type of chemotherapy, radiation dosing, and scheme or side effects of the various modalities. Lastly, due to the recency of the targeted therapies, SEER database does not give insight into their impact as a treatment option for metastatic disease.

## Conclusion

Papillary RCC is the second most common subtype of RCC. This study is one of the largest to date from the SEER database, and we include data from the COSMIC database on prevalent mutations. Distant metastasis, tumor size >10 cm, Black race, age >60 years, and undifferentiated Grade IV cancers were associated with poor survival rates. Molecular characterization of tumors will better assist in providing patients with a personalized treatment regimen.

## References

[1] Moch H, Amin MB, Berney DM, Compérat EM, Gill AJ, Hartmann A, et al. The 2022 World Health Organization classification of tumours of the urinary system and male genital organs—Part A: Renal, penile, and testicular tumours. Eur Urol. 2022;82(5):458–68. 10.1016/j.eururo.2022.06.016 35853783

[2] Mendhiratta N, Muraki P, Sisk AE Jr, Shuch B. Papillary renal cell carcinoma: Review. Urol Oncol. 2021;39(6):327–37. 10.1016/j.urolonc.2021.04.013 34034966

[3] Siegel RL, Miller KD, Fuchs HE, Jemal A. Cancer statistics., 2022. CA Cancer J Clin. 2022;72(1):7–33. 10.3322/caac.21708 35020204

[4] Lobo J, Ohashi R, Amin MB, Berney DM, Compérat EM, Cree IA, et al. WHO 2022 landscape of papillary and chromophobe renal cell carcinoma. Histopathology. 2022;81(4):426–38. 10.1111/his.14700 35596618

[5] Angori S, Lobo J, Moch H. Papillary renal cell carcinoma: Current and controversial issues. Curr Opin Urol. 2022;32(4):344–51. 10.1097/MOU.0000000000001000 35674688 PMC9394504

[6] Cornejo KM, Dong F, Zhou AG, Wu CL, Young RH, Braaten K, et al. Papillary renal cell carcinoma: Correlation of tumor grade and histologic characteristics with clinical outcome. Human Pathol. 2015;46(10):1411–7. 10.1016/j.humpath.2015.07.001 26297250

[7] Sukov WR, Lohse CM, Leibovich BC, Thompson RH, Cheville JC. Clinical and pathological features associated with prognosis in patients with papillary renal cell carcinoma. J Urol. 2012;187(1):54–9. 10.1016/j.juro.2011.09.053 22088335

[8] Akhtar M, Al-Bozom IA, Al Hussain T. Papillary renal cell carcinoma (PRCC): An update. Adv Anat Pathol. 2019;26(2):124–32. 10.1097/PAP.0000000000000220 30507616

[9] Skinner DG, Colvin RB, Vermillion CD, Pfister RC, Leadbetter WF. Diagnosis and management of renal cell carcinoma. A clinical and pathologic study of 309 cases. Cancer. 1971;28(5):1165–77. 10.1002/1097-0142(1971)28:53.0.co;2-g 5125665

[10] Abou Elkassem AM, Lo SS, Gunn AJ, Shuch BM, Dewitt-Foy ME, Abouassaly R, et al. Role of imaging in renal cell carcinoma: A multidisciplinary perspective. Radiographics. 2021;41(5):1387–407. 10.1148/rg.2021200202 34270355

[11] Chiarello MA, Mali RD, Kang SK. Diagnostic accuracy of MRI for detection of papillary renal cell carcinoma: A systematic and meta-analysis. AJR Am J Roentgenol. 2018;211(4), 812–21. 10.2214/AJR.17.19462 30063398 PMC6440798

[12] Srinivasan R, Hammerich K. Papillary renal cell carcinoma. In: Malouf G, Tannir N, editors. Rare kidney tumors. Cham: Springer; 2019. pp. 53–63.

[13] Murugan P, Jia L, Dinatale RG, Assel M, Benfante N, Al-Ahmadie HA, et al. Papillary renal cell carcinoma: A single institutional study of 199 cases addressing classification, clinicopathologic and molecular features, and treatment outcome. Mod Pathol. 2022;35(6):825–35. 10.1038/s41379-021-00990-9 34949764 PMC9177523

[14] Patel HD, Pierorazio PM, Johnson MH, Sharma R, Iyoha E, Allaf ME, et al. Renal functional outcomes after surgery, ablation, and active surveillance of localized renal tumors: A systematic review and meta-analysis. Clin J Am Soc Nephrol. 2017;12(7):1057–69. 10.2215/CJN.11941116 28483780 PMC5498358

[15] Van Poppel H, Da Pozzo L, Albrecht W, Matveev V, Bono A, Borkowski A, et al. A prospective, randomised EORTC intergroup phase 3 study comparing the oncologic outcome of elective nephron-sparing surgery and radical nephrectomy for low-stage renal cell carcinoma. Eur Urol. 2011;59(4):543–52. 10.1016/j.eururo.2010.12.013 21186077

[16] Vera-Badillo FE, Templeton AJ, Duran I, Ocana A, de Gouveia P, Aneja P, et al. Systemic therapy for non-clear cell renal cell carcinomas: A systematic review and meta-analysis. Eur Urol. 2015;67(4), 740–9. 10.1016/j.eururo.2014.05.010 24882670

[17] Choueiri TK, Heng DYC, Lee JL, Cancel M, Verheijen RB, Mellemgaard A, et al. Efficacy of Savolitinib vs sunitinib in patients with MET-driven papillary renal cell carcinoma: The SAVOIR phase 3 randomized clinical trial. JAMA Oncol. 2020;6(8):1247–55. 10.1001/jamaoncol.2020.2218 32469384 PMC7260692

[18] de Vries-Brilland M, McDermott DF, Suárez C, Powles T, Gross-Goupil M, Ravaud A, et al. Checkpoint inhibitors in metastatic papillary renal cell carcinoma. Cancer Treat Rev. 2022;99:102228. 10.1016/j.ctrv.2021.102228 34111642

[19] Martínez Chanzá N, Xie W, Asim Bilen M, Dzimitrowicz H, Burkart J, Geynisman DM, et al. Cabozantinib in advanced non-clear-cell renal cell carcinoma: A multicentre, retrospective, cohort study. Lancet Oncol. 2019;20(4):581–90. 10.1016/S1470-2045(18)30907-0 30827746 PMC6849381

[20] Pal SK, Tangen C, Thompson IM Jr, Balzer-Haas N, George DJ, Heng DYC, et al. A comparison of sunitinib with cabozantinib, crizotinib, and savolitinib for treatment of advanced papillary renal cell carcinoma: A randomised, open-label, phase 2 trial. Lancet. 2021;397(10275):695–703. 10.1016/S0140-6736(21)00152-5 33592176 PMC8687736

[21] Escudier B, Molinie V, Bracarda S, Maroto P, Szczylik C, Nathan P, et al. Open-label phase 2 trial of first-line everolimus monotherapy in patients with papillary metastatic renal cell carcinoma: RAPTOR final analysis. Eur J Cancer. 2016;69:226–35. 10.1016/j.ejca.2016.08.004 27680407

[22] Hudes G, Carducci M, Tomczak P, Dutcher J, Figlin R, Kapoor A, et al. Temsirolimus, interferon alfa, or both for advanced renal-cell carcinoma. N Engl J Med. 2016;356(22):2271–81. 10.1056/NEJMoa066838 17538086

[23] Dutcher JP, de Souza P, McDermott D, Figlin RA, Berkenblit A, Thiele A, et al. Effect of temsirolimus versus interferon-alpha on outcome of patients with advanced renal cell carcinoma of different tumor histologies. Med Oncol. 2009;26(2):202–9. 10.1007/s12032-009-9177-0 19229667

[24] Klatte T, Pantuck AJ, Said JW, Seligson DB, Rao NP, LaRochelle JC, et al. Cytogenetic and molecular tumor profiling for type 1 and type 2 papillary renal cell carcinoma. Clin Cancer Res. 2009;15(4):1162–9. 10.1158/1078-0432.CCR-08-1229 19228721

[25] Cancer Genome Atlas Research Network, Linehan WM, Spellman PT, Ricketts CJ, Creighton CJ, Fei SS, et al. Comprehensive molecular characterization of papillary renal-cell carcinoma. N Engl J Med. 2016;374(2):135–45. 10.1056/NEJMoa1505917 26536169 PMC4775252

[26] Ledezma RA, Negron E, Paner GP, Rjepaj C, Lascano D, Haseebuddin M, et al. Clinically localized type 1 and 2 papillary renal cell carcinomas have similar survival outcomes following surgery. World J Urol. 2016;34(5):687–93. 10.1007/s00345-015-1692-3 26407582

[27] Park I, Shim YS, Go H, Hong BS, Lee JL. Long-term response of metastatic hereditary leiomyomatosis and renal cell carcinoma syndrome associated renal cell carcinoma to bevacizumab plus erlotinib after temsirolimus and axitinib treatment failures. BMC Urol. 2019;19(1):51. 10.1186/s12894-019-0484-2 31182090 PMC6558845

[28] Choi Y., Keam B., Kim M., Yoon S., Kim D., Choi J. G., et al. Bevacizumab plus Erlotinib combination therapy for advanced hereditary leiomyomatosis and renal cell carcinoma-associated renal cell carcinoma: A multicenter retrospective analysis in Korean patients. Cancer Res Treat. 2019;51(4):1549–56. 10.4143/crt.2019.086 30913859 PMC6790829

[29] Schrader AJ, Rauer-Bruening S, Olbert PJ, Hegele A, Rustemeier J, Timmesfeld N, et al. Incidence and long-term prognosis of papillary renal cell carcinoma. J Cancer Res Clin Oncol. 2009;135(6):799–805. 10.1007/s00432-008-0515-y 19023595 PMC12160177

[30] Ronnen EA, Kondagunta GV, Ishill N, Spodek L, Russo P, Reuter V, et al. Treatment outcome for metastatic papillary renal cell carcinoma patients. Cancer. 2006;107(11):2617–21. 10.1002/cncr.22340 17083126

[31] Pan H, Ye L, Zhu Q, Yang Z, Hu M. The effect of the papillary renal cell carcinoma subtype on oncological outcomes. Sci Rep. 2020;10:21073. 10.1038/s41598-020-78174-9 33273677 PMC7713298

[32] Cancer Genome Atlas Research Network. Comprehensive molecular profiling of lung adenocarcinoma. Nature. 2014;511(7511):543–50. 10.1038/nature13385 25079552 PMC4231481

[33] Wolf J, Seto T, Han JY, Reguart N, Garon EB, Groen HJM, et al. Capmatinib in MET exon 14-mutated or MET-amplified non-small-cell lung cancer. N Engl J Med. 2020;383(10):944–57. 10.1056/NEJMoa2002787 32877583

[34] Camidge DR, Otterson GA, Clark JW, Ignatius Ou SH, Weiss J, Ades S, et al. Crizotinib in patients with MET-amplified NSCLC. J Thorac Oncol. 2021;16(6):1017–29. 10.1016/j.jtho.2021.02.010 33676017

[35] Choueiri TK, Heng DYC, Lee JL, Cancel M, Verheijen RB, Mellemgaard A, et al. Efficacy of savolitinib vs sunitinib in patients with MET-driven papillary renal cell carcinoma: The SAVOIR Phase 3 randomized clinical Trial. JAMA Oncol. 2020;6(8):1247–55. 10.1001/jamaoncol.2020.2218 32469384 PMC7260692

[36] Yang XJ, Tan MH, Kim HL, Ditlev JA, Betten MW, Png CE, et al. A molecular classification of papillary renal cell carcinoma. Cancer Res. 2020;65(13):5628–37. 10.1158/0008-5472.CAN-05-0533 15994935

[37] Srivastava A, Doppalapudi SK, Patel HV, Srinivasan R, Singer EA. The roaring 2020s: A new decade of systemic therapy for renal cell carcinoma. Curr Opin Oncol. 2022;34(3):234–42. 10.1097/CCO.0000000000000831 35266906 PMC9177746

[38] Ronnen EA, Kondagunta GV, Ishill N, Spodek L, Russo P, Reuter V, et al. Treatment outcome for metastatic papillary renal cell carcinoma patients. Cancer. 2006;107(11):2617–21. 10.1002/cncr.22340 17083126

